# Intrinsic Resistance Switching in Amorphous Silicon Suboxides: The Role of Columnar Microstructure

**DOI:** 10.1038/s41598-017-09565-8

**Published:** 2017-08-24

**Authors:** M. S. Munde, A. Mehonic, W. H. Ng, M. Buckwell, L. Montesi, M. Bosman, A. L. Shluger, A. J. Kenyon

**Affiliations:** 1 Department of Electrical & Electronic Engineering, University College London, Torrington Place, London, WC1E 7JE UK; 2 Institute of Materials Research and Engineering, A*STAR (Agency for Science, Technology and Research), 2 Fusionopolis Way, Innovis, #08-03, Singapore, 138634 Singapore; 3 Department of Physics & Astronomy, University College London, Gower Street, London, WC1E 6BT UK

## Abstract

We studied intrinsic resistance switching behaviour in sputter-deposited amorphous silicon suboxide (a-SiO_*x*_) films with varying degrees of roughness at the oxide-electrode interface. By combining electrical probing measurements, atomic force microscopy (AFM), and scanning transmission electron microscopy (STEM), we observe that devices with rougher oxide-electrode interfaces exhibit lower electroforming voltages and more reliable switching behaviour. We show that rougher interfaces are consistent with enhanced columnar microstructure in the oxide layer. Our results suggest that columnar microstructure in the oxide will be a key factor to consider for the optimization of future SiO*x*-based resistance random access memory.

## Introduction

Amorphous silicon suboxides (a-SiO*x*) are promising candidates as switching layers in resistance random access memories (RRAM)^1–10^, with additional applications in logic devices^11^ and neuromorphic engineering^12, 13^. a-SiO*x* has key advantages over other oxide materials since it is cheaply produced from abundant resources and readily integrable into current complementary-metal-oxide-semiconductor (CMOS) technology. The optimization of an a-SiO*x*-based RRAM device would therefore be highly desirable.

Previous studies on a-SiO*x* have reported extrinsic resistance switching from metallic filamentation as a result of ion migration from the electrode material^1–3, 14^, or intrinsic conductive path formation as a result of oxygen vacancy accumulation^4, 5, 15–18^. In the latter case, switching behaviour varies greatly between devices. For example, the devices in refs 4, 6, 15, and 19 required vacuum conditions or hermetic sealing to function, with switching only observed at an exposed oxide surface. In the remaining cases, including the present study, switching occurs in the oxide bulk in ambient conditions. Previously, the oxide layer has been fabricated using a number of different techniques, including sputtering^5, 6, 8–10, 18, 20^, plasma-enhanced chemical vapour deposition (PECVD)^7^, and thermal oxidation^4, 15^. Prior studies suggest that the oxide fabrication technique strongly influences the microstructure of a-SiO*x*. Two structural models have most commonly been proposed: the ‘random bonding model’^21–23^ and the ‘random mixture model’^24–28^. Differences in microstructure are clearly likely to affect the nature of the resistance switching behaviour; however, relatively few studies^29, 30^ have been carried out on this topic in the context of RRAM and we are not aware of such investigations for an a-SiO*x* oxide layer.

In the present study we concentrate on sputtering deposition. At low substrate temperatures, it has been noted that sputtered films exhibit columnar growth^31, 32^, resulting in columnar grain structures separated by intercolumnar boundary regions, which are less densely packed with atoms. This columnar microstructure is more well-defined if the substrate surface has a greater roughness, since particles arriving at the surface tend to aggregate at high points as a result of atomic shadowing effects^31^, with intercolumnar boundaries aligned with low points on the surface.

We combine electrical probing measurements with atomic force microscopy (AFM) and scanning transmission electron microscopy (STEM), to study resistance switching behaviour in a series of devices with varying degrees of roughness at their oxide-electrode interfaces. AFM and STEM imaging indicates that rougher oxide-electrode interfaces are associated with lower electroforming voltages and more reliable switching behaviour, and are consistent with well-defined columnar microstructure in the oxide. Our results suggest that columnar microstructure is a key factor to consider for the optimization of a-SiO*x*-based RRAM technology.

## Results

**Figure 1:**
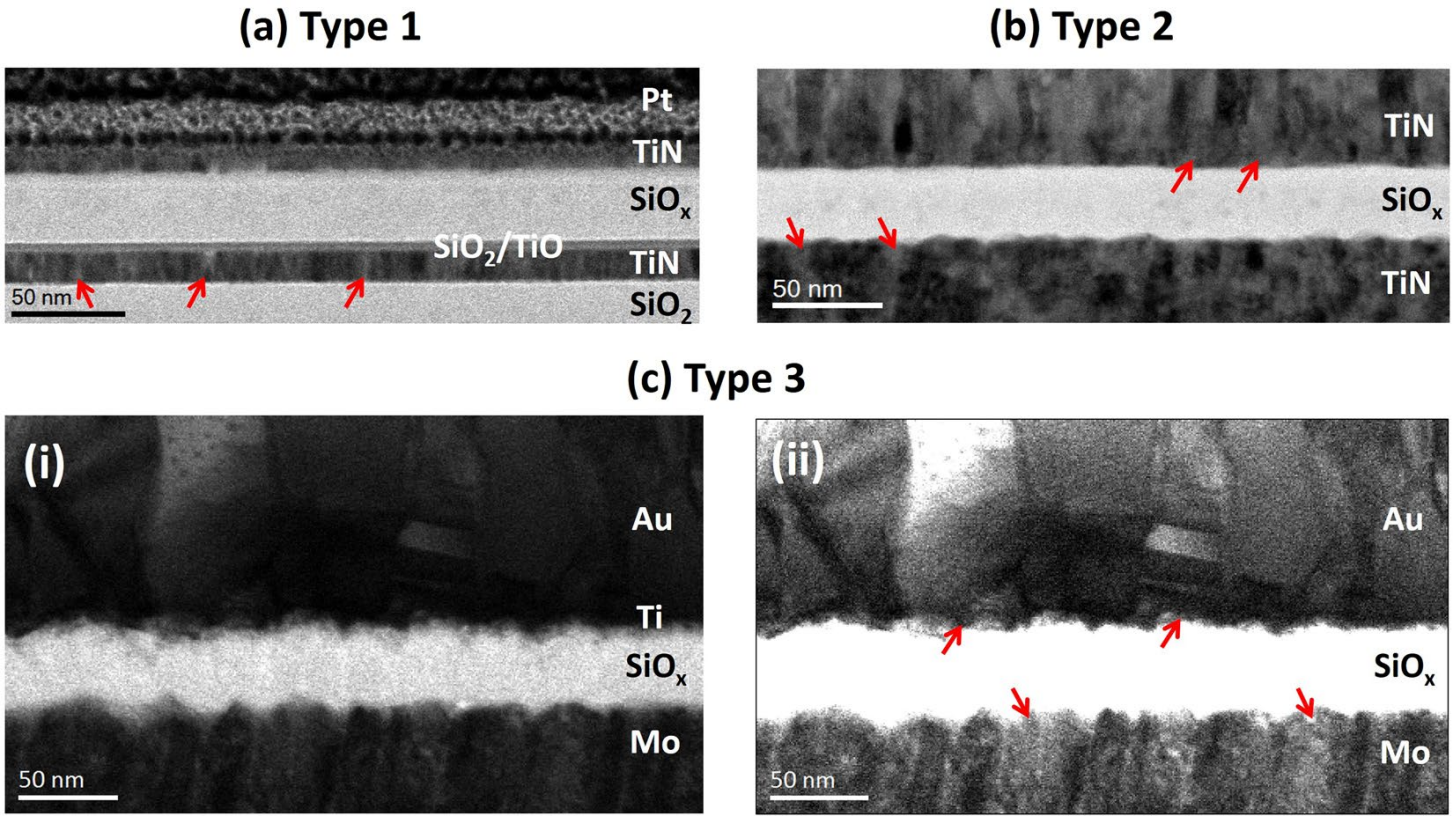
Bright field STEM images of cross-sections from type 1, type 2, and type 3 devices, with red arrows pointing out columnar grains visible in the electrode layers. The top Pt layer seen in type 1 devices is needed for focused ion beam (FIB) sample preparation. (**a**) Type 1 devices consist of TiN top and bottom electrodes, which are approximately 10 and 15 nm thick, respectively. The oxide layer is approximately 35 nm thick. A sub-5 nm mixing layer is visible at the bottom electrode. Its characteristics were determined using EELS. (**b**) Type 2 devices consist of TiN top and bottom electrodes, which are approximately 85 and 80 nm thick, respectively. The oxide layer is approximately 35 nm thick. (**c**) Type 3 devices consist of Au and Mo top and bottom electrodes, which are approximately 115 and 280 nm thick, respectively. The oxide layer is approximately 35 nm thick. A thin Ti wetting layer is present between the Au electrode and oxide layer. Its presence and approximate thickness of 5 nm was confirmed using EELS. (**c**)(ii) is a contrast-enhanced copy of (**c**)(i) and indicates columnar growth in the electrode layers.

**Figure 2:**
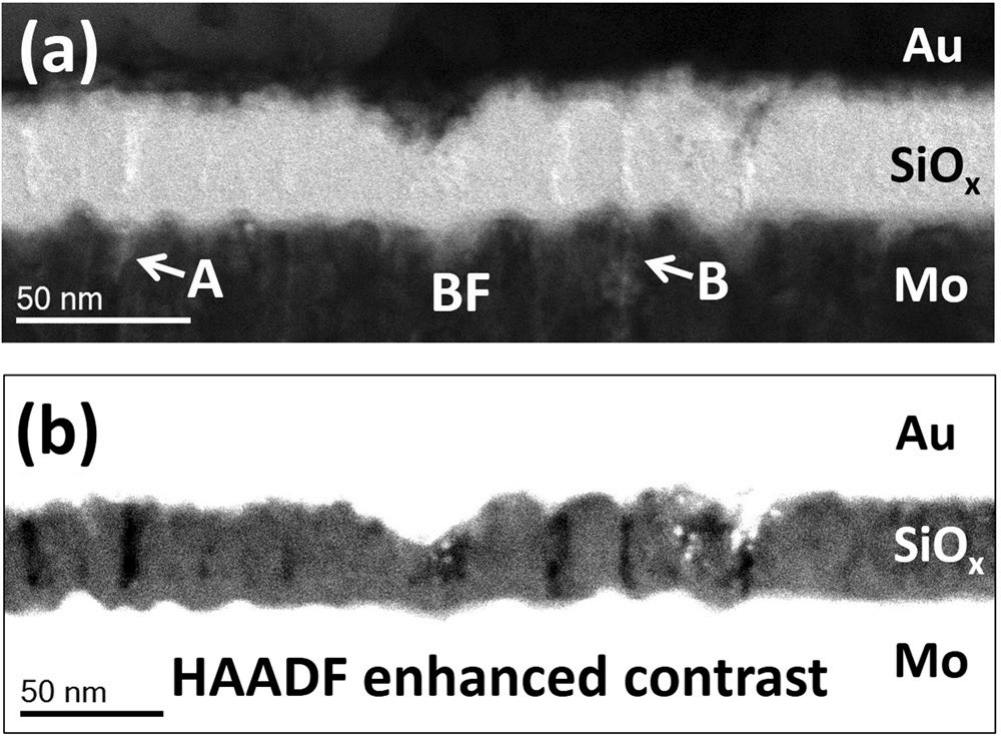
Bright and dark field STEM images of a cross-section of a type 3 device. This figure is a slightly modified version of Fig. 4 from ref. 10. (**a**) Bright field image showing that at features A and B, columnar boundaries in the Mo layer appear to extend into the oxide layer in the form of bright vertical streaks. These streaks are visible across the oxide layer with a spacing of approximately 20 nm. (**b**) Contrast-enhanced high-angle annular dark field (HAADF) image corresponding to (**a**). The bright vertical streaks in (**a**) can be seen to correspond to regions of low intensity in (**b**). This indicates regions of low average atomic number^35^ and suggests the presence of intercolumnar boundaries in the oxide layer, which are less densely packed with atoms.

Initially, three different metal-insulator-metal (MIM) devices were studied, which will be referred to as type 1, type 2, and type 3, respectively. The structures of these devices are summarized in Fig. 1. In all three devices, columnar microstructure is visible in the electrode layers as indicated by the red arrows, with increasing roughness at the bottom electrode in the order 1 < 2 (RMS roughness = 0.55 nm) < 3 (RMS roughness = 1.10 nm). In the type 3 device, columnar growth appears to be continuous through the thickness of the bottom electrode. In comparison, the top electrode appears to consist of grains stacked above one another to form relatively wider columns. It is well known that the physical properties of the deposited material greatly influence the resulting film microstructure^33, 34^. Increasingly rough bottom electrode surfaces would suggest enhanced atomic shadowing during sputter-deposition of the oxide, and more pronounced columnar microstructure within the oxide layer. A closer inspection of the type 3 device reveals columnar growth within the oxide layer as shown in Fig. 2. Figure 2(a) shows a bright field (BF) STEM image, where columnar boundaries between grains in the bottom Mo layer at features A and B appear to extend into the oxide layer in the form of bright vertical streaks. These streaks are visible across the oxide layer with a spacing of approximately 20 nm and correspond to regions of low intensity in the high-angle annular dark field (HAADF) image in Fig. 2(b). This indicates that they are regions of low average atomic number^35^ and suggests the presence of intercolumnar boundaries in the oxide layer^31^, which are less densely packed with atoms. The occurence of atomic shadowing across the whole oxide layer is indicated by the roughness at the top oxide-electrode interface, which mirrors changes at the bottom oxide-electrode interface. Such regions are not visible in type 1 and type 2 devices; however, the less rough interfaces could suggest the presence of a less well-defined columnar microstructure, which is not easily observed through the thickness of the TEM sample. We note that although a different choice of electrodes is used in type 3 devices, our previous studies have indicated that switching is still intrinsic to the oxide layer in all three devices^5, 9, 10^. We hypothesise that the following differences in switching behaviour will therefore be strongly be influenced by the differences in the degree of columnar microstructure in the oxide, which is a product of bottom electrode roughness.

**Figure 3:**
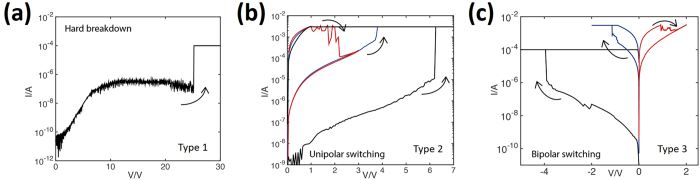
Typical I-V curves for type 1, type 2, and type 3 RRAM devices. (**a**) Type 1 devices do not electroform and only exhibit hard breakdown at around 25 V. (**b**) Type 2 devices exhibit unipolar switching behaviour, with electroforming at around 6 V, and setting and resetting at approximately 4 V and 2 V, respectively. (**c**) Type 3 devices exhibit bipolar switching behaviour, with electroforming at around −4 V, and setting and resetting at approximately −1 V and 1.5 V, respectively.

Figure 3 shows typical I-V curves obtained from the three devices. More detailed studies of the electrical characteristics of type 2 and type 3 devices are provided in refs 9 and 10. Firstly, we sweep the voltage bias to electroform (transition from the pristine state to the low resistance state) and then proceed to cyclically reset (transition from the low resistance state to the high resistance state), and set (transition from the high resistance state to the low resistance state) the device. Type 1 devices do not electroform and consistently exhibit hard breakdown (transition to a permanent low resistance state) at comparatively high voltages (≈25 V in Fig. 3(a)). Type 2 devices electroform at around 6 V and switch in a unipolar manner, with the set at around 4 V, and the reset at around 2 V (Fig. 3(b)). Despite an identical choice of electrodes and oxide layer thickness and stoichiometry in type 1 and type 2 devices, only type 2 devices electroform at a comparatively low voltage. We attribute this to the change in bottom electrode roughness, which will influence the degree of columnar microstructure in the oxide during sputtering deposition^31^. The best switching properties are obtained from type 3 devices, which have the roughest oxide-electrode interfaces. Such devices electroform at around −4 V and exhibit bipolar switching with low switching voltages, typically lower than −1 V to set, and up to 1.5 V to reset (Fig. 3(c)). In addition to having lower switching voltages than type 2 devices, type 3 devices also exhibit greater device endurance on the order of 10^7^ switching cycles^10^ as compared to 10^2^ for type 2 devices^9^. However, it should be noted that a difference in switching mechanism for unipolar and bipolar devices may greatly impact switching voltages and device endurance.

**Figure 4:**
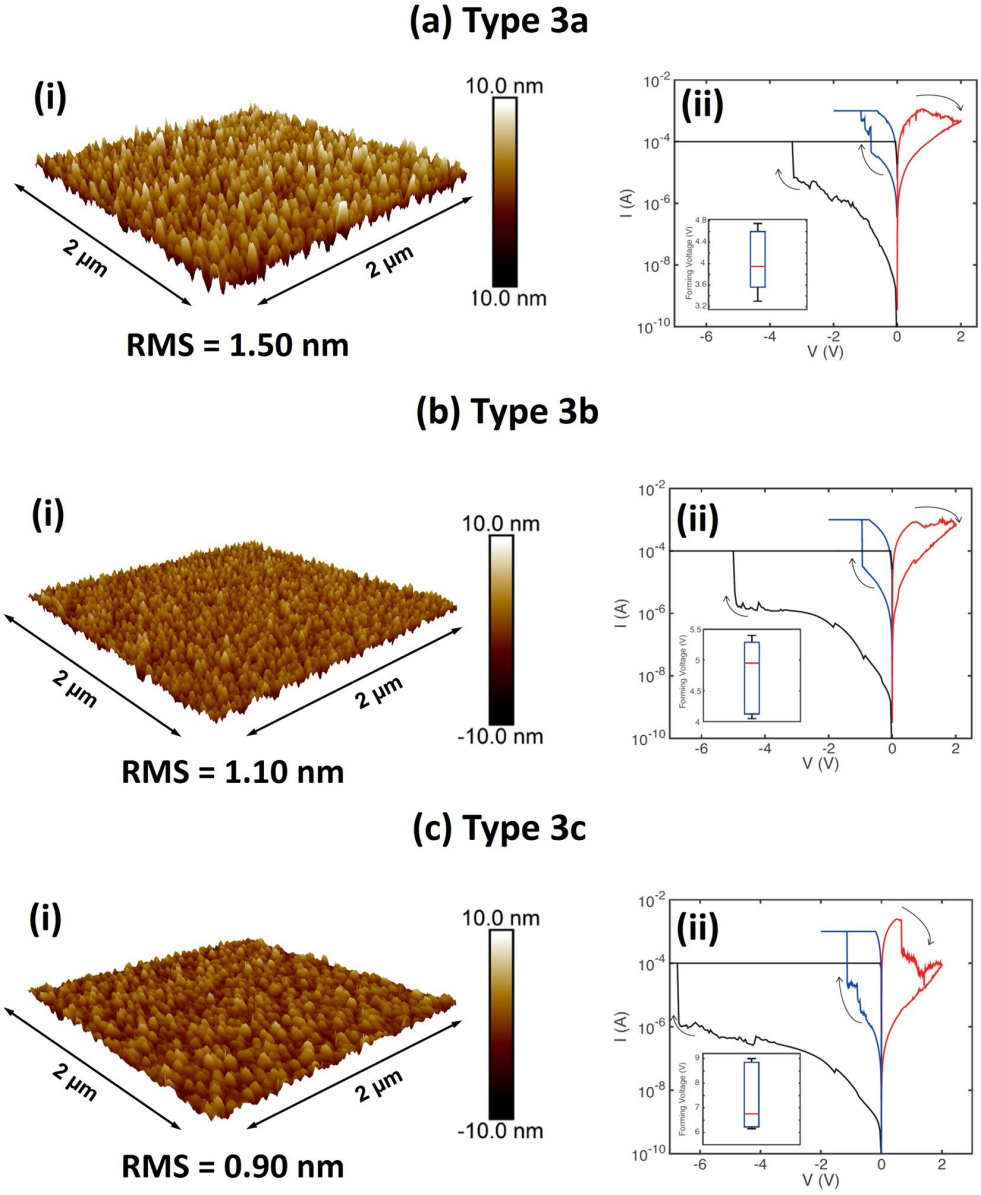
(**i**) AFM surface roughness characterization and (**ii**) Typical I-V curves for type 3 RRAM devices with varying bottom Mo electrode roughness. The insets of the I-V curves show distributions of the electroforming voltages from a sample of 5 devices for each roughness value respectively. The line inside the box is the median value, the top and bottom of the box represent the 25th and 75th percentiles, and the two whiskers represent maximum and the minimum values. (**a**) Type 3a devices electroform around a median value of 4 V, with a range between 3.3 V and 4.7 V. (**b**) Type 3b devices electroform around a median value of 5 V, with a range between 4.1 V and 5.4 V (**c**) Type 3c devices electroform around a median value 6.5 V, with a range between 6.2 V and 9.0 V. Such devices are typically less reliable, with around half of the devices failing to reset after electroforming. The reset curve (red) reveals a higher current in the low resistance state when compared to the rougher devices.

In order to isolate the effects of columnar microstructure and ensure the differences in choice of electrode layer, switching mechanism, and stoichiometry do not affect the observed changes, three type 3 devices with roughness varied at the bottom Mo electrode were fabricated. AFM topography maps for these devices are shown in Fig. 4. Type 3a, 3b, and 3c devices have an RMS roughness of 1.50 nm, 1.10 nm, and 0.90 nm, respectively, with corresponding median values for electroforming voltage around 4 V, 5 V, and 6.5 V. In addition, type 3c devices were found to be unreliable, with around half of the devices failing to reset after electroforming (see Fig. S1). As in the comparison between type 1 and type 2 devices, we again attribute this behaviour to the difference in bottom electrode roughness and the resulting columnar microstructure in the oxide. As can be observed from the reset curve in Fig. 4(c)(ii), for type 3c devices the current in the low resistance state is greater relative to the rougher devices. We suggest that this is the result of increased degradation of the oxide layer due to higher electroforming voltages, resulting in a more prominent conductive path in the low resistance state. We also note that higher electroforming voltages in type 3c devices tended to be followed by a failed reset. These results suggest that increasingly rougher oxide-electrode interfaces result in lower electroforming voltages and more reliable devices. The rougher bottom electrode would promote a greater degree of atomic shadowing during oxide deposition, and a more well-defined columnar microstructure.

## Discussion

In previous work, we measured circular dome features approximately 10 nm in diameter using an atomic force microscope (AFM)^5, 20^. Scanning tunneling microscopy (STM) revealed that these structures have increased conductivity at their edges. It was suggested that these dome structures may reflect columnar microstructure within the oxide layer, and that column boundaries may act as centers for oxygen vacancy accumulation. Analogies with this model can be drawn with resistance switching in HfO_2_ layers, where conductive paths have been shown to preferentially form at grain boundaries^36–39^. Column boundaries could be defect-rich and promote the formation of interstitial O anions^18, 40, 41^ through mechanisms such as electron injection, as suggested by Gao *et al*.^41, 42^. In this mechanism, double electron trapping at intrinsic precursor sites facilitates the formation of an oxygen vacancy and O^2−^ ion^43, 44^. More well-defined columnar boundaries, which extend across the oxide layer from the bottom electrode to the top electrode, may act as pre-defined regions for conductive path formation. This could explain the lower electroforming voltages and increased reliability recorded for rougher oxide-electrode interfaces. We hypothesise that less densely packed intercolumnar boundaries could act as low energy pathways for the transport of large quanitities of oxygen, resulting in electrode deformation and oxygen gas emission during electrical stressing as observed in previous work^7, 18, 45^.

In summary, our measurements suggest that the sputtering of a-SiO_*x*_ onto rough electrode surfaces results in lower electroforming voltages and greater device reliability for resistance switching applications. This is consistent with enhanced columnar microstructure in the oxide layer. These observations will be important to consider for the optimization of future a-SiO_*x*_ -based RRAM devices.

## Methods

Three different MIM stacks were initially studied, and are referred to as type 1, type 2, and type 3. For all device types, the metal and oxide layers were deposited by sputtering onto SiO_2_ substrates, except in type 3 devices where the top electrode layers were deposited using electron beam evaporation. Type 1 and type 2 devices were symmetrical TiN/SiO_*x*_/TiN stacks with fabrication aiming for a stoichiometry of *x* ≈ 1.3, whereas type 3 devices were non-symmetrical Au/SiO_*x*_/Mo stacks with a Ti wetting layer approximately 5 nm thick between the Au and SiO_*x*_ layers. The oxide layer in type 3 devices was comparatively oxygen-rich with fabrication aiming to achieve a stoichiometry with *x* ≈ 2. These differences in stoichiometry were confirmed from the corresponding ELNES fingerprints of the oxide^46^.

In order to isolate the effect of bottom electrode roughness (and therefore columnar microstructure) on switching behaviour, three variants of type 3 devices (type 3a, 3b, and 3c) were prepared, with different roughnesses for the bottom Mo electrode. The variation in roughness was achieved by adjusting the Mo sputtering parameters such as substrate temperature, deposition pressure, and power as described in the following: type 3a: power = 200 W, deposition pressure = 10 mTorr, substrate temperature = 25 C; type 3b: power = 300 W, deposition pressure = 5 mTorr, substrate temperature = 25 C; type 3c: power = 300 W, deposition pressure = 3 mTorr, substrate temperature = 150 C. The 35 nm a-SiO _*x*_ layer was deposited by reactive sputtering using a silicon target in an oxygen-rich environment. The Ti/Au top contact was deposited by electron beam evaporation with individual device size defined using a shadow mask.

Roughness values for the SiO_*x*_ layers were calculated from atomic force microscopy measurements. These measurements were made using a Bruker Icon microscope with a Nanoscope V controller running Nanoscope v9.1. Root mean square roughness values were calculated using Nanoscope Analysis v1.50.

All devices types were electrically stressed using a Keithley 4200 semiconductor characterization system with a Signatone probe station. All electrically stressed devices were approximately 400 *μ*m × 400 *μ*m in area.

An FEI Helios focused ion beam (FIB) was used to prepare TEM cross-sections of unstressed and stressed devices. STEM imaging was carried out at 80 KeV using an FEI Titan (S)TEM.

## Electronic supplementary material

Intrinsic Resistance Switching in Amorphous Silicon Suboxides: The Role of Columnar Microstructure
