# Supplementary material for: Intrinsic Resistance Switching in Amorphous Silicon Suboxides: The Role of Columnar Microstructure
Source: Sci Rep. 2017 Aug 24;7:9274. doi: 10.1038/s41598-017-09565-8 (PMC5571160; doi:10.1038/s41598-017-09565-8)
Supplement: Supplementary file 1 — Intrinsic Resistance Switching in Amorphous Silicon Suboxides: The Role of Columnar Microstructure [file 41598_2017_9565_MOESM1_ESM.pdf]

Intrinsic Resistance Switching in Amorphous Silicon Suboxides: The Role of Columnar Microstructure

M. S. Munde[1,2,3*], A. Mehonic[1], W. H. Ng[1], M. Buckwell[1], L. Montesi[1], M. Bosman[2], A. L. Shluger[3], and A. J. Kenyon[1]

[1]Department of Electrical & Electronic Engineering, University College London, Torrington Place, London, WC1E 7JE
[2]Institute of Materials Research and Engineering, A*STAR (Agency for Science, Technology and Research), 2 Fusionopolis Way, Innovis, #08-03, Singapore, 138634
[3]Department of Physics & Astronomy, University College London, Gower Street, London, WC1E 6BT
*manveer.munde.13@ucl.ac.uk

Supplementary Information

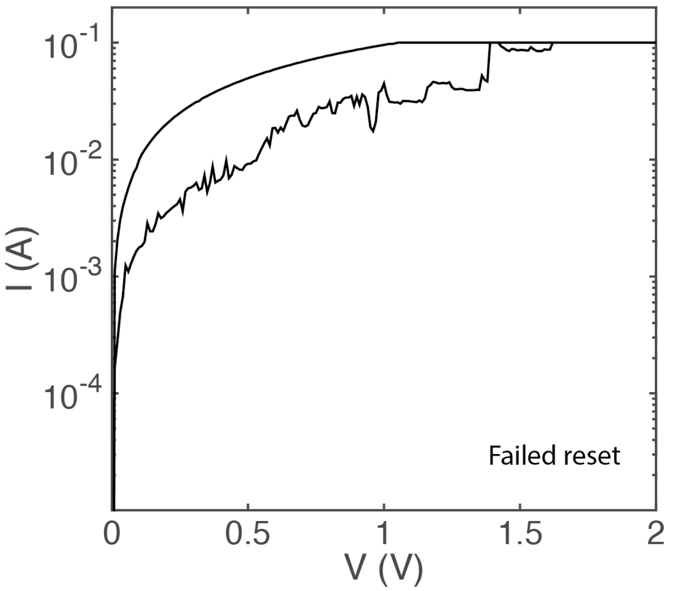

Figure S1. Example of a failed reset operation for a type 3c device after initial electroforming.
